# Uncovering the genetic mechanisms regulating panicle architecture in rice with GPWAS and GWAS

**DOI:** 10.1186/s12864-021-07391-x

**Published:** 2021-01-28

**Authors:** Hua Zhong, Shuai Liu, Xiaoxi Meng, Tong Sun, Yujuan Deng, Weilong Kong, Zhaohua Peng, Yangsheng Li

**Affiliations:** 1 State Key Laboratory of Hybrid Rice, Key Laboratory for Research and Utilization of Heterosis in Indica Rice, Ministry of Agriculture, College of Life Sciences, Wuhan University, Wuhan, China; 2 Department of Biochemistry, Molecular Biology, Entomology and Plant Pathology, Mississippi State University, Starkville, MS 39762 USA; 3 Department of Computer Science and Engineering, Experimental Teaching Center, Shijiazhuang University, Shijiazhuang, Hebei China

**Keywords:** Rice, Panicle architecture, GPWAS, GWAS

## Abstract

**Background:**

The number of panicles per plant, number of grains per panicle, and 1000-grain weight are important factors contributing to the grain yield per plant in rice. The Rice Diversity Panel 1 (RDP1) contains a total of 421 purified, homozygous rice accessions representing diverse genetic variations within *O. sativa*. The release of High-Density Rice Array (HDRA, 700 k SNPs) dataset provides a new opportunity to discover the genetic variants of panicle architectures in rice.

**Results:**

In this report, a new method genome-phenome wide association study (GPWAS) was performed with 391 individuals and 27 traits derived from RDP1 to scan the relationship between the genes and multi-traits. A total of 1985 gene models were linked to phenomic variation with a *p*-value cutoff of 4.49E-18. Besides, 406 accessions derived from RDP1 with 411,066 SNPs were used to identify QTLs associated with the total spikelets number per panicle (TSNP), grain number per panicle (GNP), empty grain number per panicle (EGNP), primary branch number (PBN), panicle length (PL), and panicle number per plant (PN) by GLM, MLM, FarmCPU, and BLINK models for genome-wide association study (GWAS) analyses. A total of 18, 21, 18, 17, 15, and 17 QTLs were identified tightly linked with TSNP, GNP, EGNP, PBN, PL, and PN, respectively. Then, a total of 23 candidate genes were mapped simultaneously using both GWAS and GPWAS methods, composed of 6, 4, 5, 4, and 4 for TSNP, GNP, EGNP, PBN, and PL. Notably, one overlapped gene (*Os01g0140100*) were further investigated based on the haplotype and gene expression profile, indicating this gene might regulate the TSNP or panicle architecture in rice.

**Conclusions:**

Nearly 30 % (30/106) QTLs co-located with the previous published genes or QTLs, indicating the power of GWAS. Besides, GPWAS is a new method to discover the relationship between genes and traits, especially the pleiotropy genes. Through comparing the results from GWAS and GPWAS, we identified 23 candidate genes related to panicle architectures in rice. This comprehensive study provides new insights into the genetic basis controlling panicle architectures in rice, which lays a foundation in rice improvement.

**Supplementary Information:**

The online version contains supplementary material available at 10.1186/s12864-021-07391-x.

## Background

Rice is an important cereal for people in 39 countries around the world, particularly in Asia, Latin America, and parts of Africa. In Asia alone, 2.7 billion people take rice as their staple food [1]. Yield increasing is an important goal in the rice improvement process, which could be divided into three major components - number of panicles per plant, number of grains per panicle, and 1000-grain weight [2]. Besides, the yield is also correlated to plant height, panicle length, seed setting rate, *etc* [3]. Several genes have been reported regulating the grains per panicle in rice, including *Gn1a* [4], *GNP1* [5], *GAD1* [6], *An-1* [7], *OsCBL8* [8], *OsDim1* [8], *OsMADS18* [9], *PAY1* [10], and *SAPK2* [11]. Gibberellins (GAs) and cytokinins play antagonistic roles in modulating the activity of the reproductive meristem. Up-regulated cytokinin activity leads to increased grain number, while GAs negatively affects meristem activity. The *Gn1a* encodes cytokinin oxidase/dehydrogenase, which can degrade the phytohormone cytokinin. Decreased expression of *OsCKX2* will lead to the accumulation of cytokinins in inflorescence meristems, and increase of the number of reproductive organs, resulting in an increased grain yield [4]. Whereas the *GNP1* is involved in GA biosynthesis. When the *GNP1* was up-regulated, the activity of cytokinin was increased because of a KNOX-mediated transcriptional feedback loop, resulting in an increased grain number and grain yield in rice [5]. Moreover, *OsOAT* [12] and *LSSR1* [13] are two reported genes regulating the seed setting rate in rice. *OsOAT* is associated with floret development and seed setting rate in rice [12] and *LSSR1* [13] regulates seed setting rate through enhancing fertilization in rice. *SD1* is also a GA biosynthetic gene, regulating plant height through manipulating the level of gibberellin in plants [14]. *OsSPL16* could increase the yield by promoting grain filling in rice [15]. *GS3* is a major gene regulating the grain size in rice grain [16], which has been used to improve yield with the CRISPR-Cas9 system [17].

Genome-wide association study (GWAS) is an efficient method to map QTLs or genes related to target traits and has been successfully applied in many plants, including *Arabidopsis* [18], maize [19], soybean [20], rice [21], *etc*. The general linear model (GLM) and mixed linear models (MLM) are two univariate models to perform GWAS analysis that have successfully mapped several trait-associated genes [22, 23]. Alternative methods such as Fixed and random model Circulating Probability Unification (FarmCPU) [24] and Bayesian-information and Linkage-disequilibrium Iteratively Nested Keyway (BLINK) [25] are multivariate methods to reduce the false-positive rate and increase the statistic power. A previous study reported that using the univariate and multivariate in a combined way could increase the mapping efficiency of QTLs [26].

As a novel algorithm, Genome-phenome wide association study (GPWAS) has been developed to identify the links between genes and quantitative phenotypic variations via a multi-trait multi-SNP framework [27]. In general, GWAS scans the variation of the SNPs correlated to the divergence of target traits, while GPWAS is a reverse process compared to GWAS. GPWAS uses phenotypic traits as a matrix and evaluates the relationship between multi-trait and gene models and then the best-fit gene model was selected associated with the multiple phenotypes. GPWAS has been successfully applied in *Arabidopsis* and maize [27], but no reported in rice by far.

The Rice Diversity Panel 1 (RDP1) contains a total of 421 purified, homozygous rice accessions representing a diverse genetic variation within *O. sativa* [28]. Fifteen yield-related traits have been studied in RDP1 with MLM using GWAS, and more than 100 overlapped QTLs were selected via comparing the GWAS results with bi-parental populations [29]. In this study, through a combination of GWAS and GPWAS methods, we identified QTLs or genes associated with TSNP, GNP, EGNP, PBN, PL, and PN. Besides, the overlapped gene *Os01g0140100* was further investigated by haplotype analysis based on non-synonymous SNPs. Haplotype C (Pro4Ser501) exhibits the highest number of spikelets and grain number per panicle among the four types of haplotypes. Moreover, gene expression patterns manifested that this gene is highly expressed in pre-emergence inflorescence, seed-5 DAP (seed 5 days after pollination), and pistil tissues. We suggested that *Os01g0140100* is a candidate gene linked to the yield by regulating the florets per panicle in rice.

## Results

### Population structure and linkage disequilibrium

**Fig. 1:**
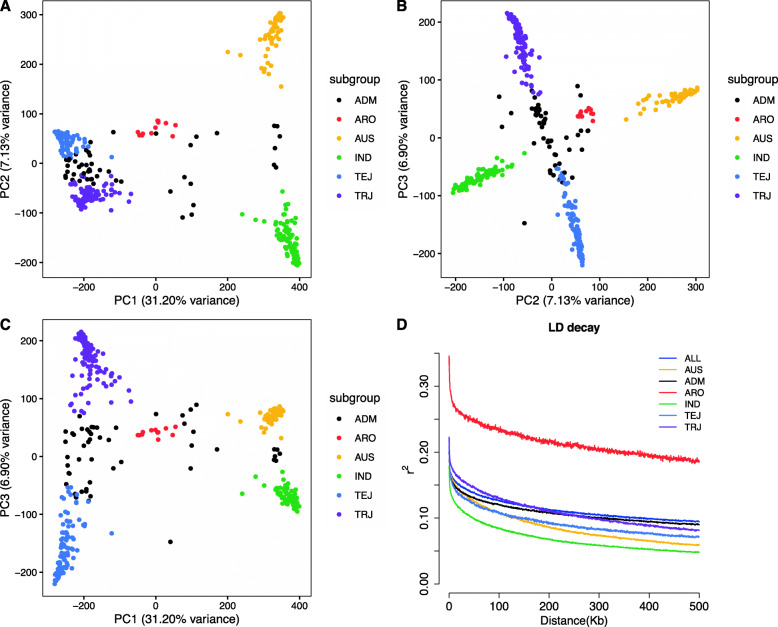
Genetic structure of the rice diversity panel 1. Principal component analysis shows the genetic variation in the rice accessions with (**a**) first and second principal components (PCs), (**b**) second and third PCs, and (**c**) first and third PCs. (**d**) Genome-wide average linkage disequilibrium decay estimated of the whole population and subpopulations

Principal component analysis (PCA) was performed based on the 411,066 SNPs. Five conceivable subpopulations were separated by the first three principal components (PCs) (Fig. 1a-c), explaining over 45% of the genetic variation. The PC1 separated IND, *Japonica* (TEJ and TRJ), and ARO subpopulations (31.20%), the PC2 (7.13%) distinguished the AUS and IND varieties, and the PC3 (6.90%) separated TEJ and TRJ varietal subgroups. Moreover, the kinship analysis (Supplementary Fig. 1) was performed to study the relativeness between the varieties. The results exhibited that the population could be divided into five groups (the red line in Supplementary Fig. 1), which was corresponding to the PCA analysis. The linkage disequilibrium (LD) decay of the physical distance between SNPs in all populations occurred at 250 kb when the pairwise coefficient of determination dropped to half of its maximum value (*r*^2^ = 0.107). The IND subgroup exhibited the most rapid LD decay and ARO displayed the most extended LD (Fig. 1d).

### Genome-Phenome Wide Association Study (GPWAS)

Based on the filtering criteria in the method section, 23,623 of 37,860 annotated genes containing 95,324 SNPs were left for the GPWAS study. The majority of genes (22,233, 94.12%) possessed less than 10 SNPs, while 1390 (5.88%) genes harbored more than 10 SNPs (Supplementary Fig. 2). The genome-phenome wide association study was performed by GPWAS software (https://github.com/shanwai1234/GPWAS). The detailed information of 1985 selected genes related to TSNP, GNP, EGNP, PBN, PL, or PN was listed in Supplementary Table 2. Based on the permutation analysis, a *p*-value cutoff was 4.49E-18, resulting in an estimated FDR of < 2.00E-3.

Notably, the most significant gene (*OsROS1*, *Os01g0218032*) with a *p*-value of 3.79E-59 encodes a DNA demethylase that could remove 5-methylcytosine. This locus was identified associated with 12 traits including EGNP, HULGRLGWDRO, CULMHAB, PTHT, FLFLG, FLFWD, PL, DTHD, TSNP, GNP, PN, and PBN. A previous study reported that loss-of-function mutations of this gene showed sterile or with extremely low seed setting rate at around 6% [30]. Sterile or lower grain setting rate is related to EGNP, DTHD, TSNP, and GNP, which was consistent with our GPWAS study. Another gene (*OsCOM1*, *Os06g0613400*) was detected to be associated with DHULGRWD, PTHT, EGNP, TSNP, GNP, FLFWD, LFLPUBES, PN, HHULGRWT, and CULMHAB traits with a significant *p*-value of 1.10E-50. *OsCOM1* plays a key role in regulating recombination in rice meiosis [31]. *Oscom1–1* mutant caused entangled chromosome mass in metaphase I, producing unequal segregation of chromosomes to the two daughter cells anaphase I, resulting in a sterile architecture. This phenotype was also related to the EGNP, GNP, *etc*. The *Ghd7.1* (*Os07g0695100*) was proved to be related to spikelet per panicle, plant height, and heading date in rice [32], and this gene was identified associated with PTHT, PBN, GNP, DHULPROTCN, HULGRLGWDRO, LFLPUBES, AMYCN with the *p*-value of 1.41E-20. All of the above genes demonstrated the statistical power of the GPWAS method.

### Genome-Wide Association Study (GWAS)

**Fig. 2:**
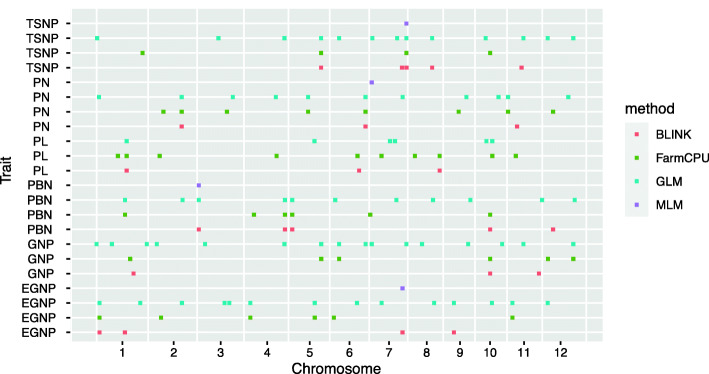
QTLs associated with six traits of total spikelets number per panicle (TSNP), empty grain number per panicle (EGNP), grain number per panicle (GNP), primary branch number (PNB), panicle length (PL), and panicle number per plant (PN) with four different methods

All the six traits were analyzed using two univariate GWAS (GLM and MLM) and two multivariate GWAS (FarmCPU and BLINK) methods to identify QTLs. The PCA matrix was used in the GLM approach to correct the population structure. Both the PCA and relatedness matrixes were incorporated in the MLM model to reduce the false-positive rate. FarmCPU uses both the fixed-effect model and the random effect model iteratively to control false positives and avoid the over-fitting problem. BLINK approximates the maximum likelihood using Bayesian Information Content (BIC) in a fixed-effect model to reduce the amount of calculation. All the detailed Manhattan and quantile-quantile figures were depicted in Supplementary Figs. 3, 4, 5, 6, 7, and 8. Using the threshold mentioned in method, 18, 21, 18, 17, 15, and 17 QTLs were identified associated with TSNP, GNP, EGNP, PNB, PL, and PN, respectively (Fig. 2).

Among the 18 unique loci correlated with TSNP tightly, 13, 1, 4, and 5 QTLs were detected by GLM, MLM, FarmCPU, and BLINK method, respectively (Supplementary Table 3 and Supplementary Fig. 3). S4_30920978 was located 18.19 kb away from the previously reported gene *OsERF77* (ETHYLENE RESPONSE FACTOR 77), detected only using the GLM method. Overexpression of *OsERF77* led to a reduction in yield by decreasing the biomass and the number of seeds in the transgenic lines [33]. The S5_23732692 was a significant QTL located on chromosome 5 identified by three methods, including GLM, FarmCPU, and BLINK. This locus is located at the reported gene *EUI1* (ELONGATED UPPERMOST INTERNODE 1), encoding a putative cytochrome P450 monooxygenase and specifically expressed in young panicles [34]. *EUI1* was proved to be a key regulator controlling the elongation of the uppermost internode in rice during the heading stage using overexpression and RNAi technology [34] and a recessive rice *eui* could increase panicle length [35]. Although there was no study reported the relationship between *EUI1* and total spikelets number per panicle, we suggested that *EUI1* was a candidate gene regulating spikelets per panicle from GWAS analyses, which could be explained by extending the length of panicles. Another QTL S6_7637690 located on chromosome 6 was detected solely by the GLM method with a *p*-value of 5.25E-10. This locus was only 11.30 kb away from *OsPFPB*. *OsPFPB* contains a pyrophosphate-fructose 6-phosphate 1-phosphotransferase (PFP) beta subunit, which plays a key role in carbon metabolism during rice grain filling. A defective endosperm mutant *pfp1–3* showed a slower grain-filling rate, reduced 1000-grain weight, decreased number of panicles per plant, lower plant height, and declined grain yield per plant [36]. Nevertheless, no significant difference was detected in grain number per panicle [36]. The S7_28205415 locus was detected by GLM, MLM, and FarmCPU methods with the *p*-value of 2.16E-12, 6.54E-08, and 1.13E-09, respectively. Besides, this was the only QTL detected with MLM function. This locus located at the 28,205,415 bp on chromosome 7 and a previous reported gene *OsCOL13* posits 17.57 kb downstream of the QTL. The *OsCOL13* gene is a CONSTANS-like transcriptional activator and negatively mediates the flowering in rice through manipulating *OsphyB* and *Ehd1* [37]. Another QTL S7_28316717 was only detected by the BLINK method. This locus lied on 17.12 kb downstream of a reported gene *FZP* [38]. The *FZP* gene encodes an ERF (ethylene-responsive factor) transcription factor, which regulates the transition from spikelet to floret meristem. The loss-of-function mutant showed abnormal on developing florets without impacting primary rachis-branches [38]. Moreover, three QTLs were reported in the bi-parental population covering the region of *OsERF77*, *EUI1*, and *OsPFPB* gene, respectively (Supplementary Table 3). Another QTL *qSPBp10–2* was reported associated with number of spikelets on primary branches per panicle on chromosome 10, which was located in the same physical region of S10_11692566. In addition, nine novel QTLs linked with TSNP were identified by GLM. One unique QTLs (S1_37112077) were detected by FarmCPU. Three new loci (S7_24847205, S8_18445162, and S11_12703414) associated with TSNP were discovered by BLINK.

A total of 21 unique QTLs were picked out to be associated with GNP. Sixteen, six, and three QTLs were detected by GLM, FarmCPU, and BLINK method, respectively (Supplementary Table 3 and Supplementary Fig. 4). No loci were discovered by MLM due to the overcorrection with PCA and kinship matrix. Five loci (S4_30920978, S5_23732692, S6_7637690, S7_28208986, and S6_28075474) were mapped to previously reported genes (*OsERF77*, *EUI1*, *OsPFPB*, *OsCOL13,* and *DEP3* [33, 34, 36, 37, 39] related to seed, panicle, or flowering development. A previously reported gene *DEP3* is located 33.76 kb downstream of the S6_28075474. The *DEP3* encodes a patatin-related phospholipase A, playing a key role in regulating panicle length, grain shapes, and grain number per panicle [39]. Four new loci (S1_27584958, S10_11692566, S12_4022340, and S12_23573438) were identified by the FarmCPU method, two (S1_27584958 and S12_4022340) of which were mapped by FarmCPU algorithm exclusively. The S10_11692566 was detected by BLINK and S12_23573438 was identified by the GLM method as well. Also, two more novel loci (S1_30211939 and S11_26117686) were discovered by BLINK.

Among the 18 unique QTLs associated with EGNP, 14, 1, 6, and 4 loci were discovered by GLM, MLM, FarmCPU, and BLINK methods, respectively (Supplementary Table 3 and Supplementary Fig. 5). A QTL (S3_24818570) located on chromosome 3 was identified by GLM with a *p*-value of 1.32E-09. This locus stood on merely 0.53 kb downstream of an Ornithine δ-aminotransferase gene named as *OsOAT*. *OsOAT* has an impact on reutilizing nitrogen through regulating arginase activity and the loss-of-function mutation exhibits abnormal seed shape and lowers seed setting rate [12]. S4_4674594 was another locus related to EGNP recognized by GLM and FarmCPU methods. This QTL was located in the 4,674,594 bp on chromosome 4, about 67.18 kb away from *OsBOR4*. *OsBOR4* is an active efflux transporter of boron, which regulating pollen germination and tube elongation in rice [40]. Under the deficiency of boron, the mutant *osbor1–1* plants showed significantly smaller architecture with immature seeds compared to the wild-type at the mature period [41]. Besides, four QTLs (S1_35375938, S2_8170035, S6_3547612, and S11_5603525) were also reported associated with spikelet fertility in the Q-TARO database (http://qtaro.abr.affrc.go.jp/) (Supplementary Table 3). Moreover, eight novel QTLs related to EGNP were identified by GLM. Among them, S5_18792970, S7_25286267, and S9_6774209 were also mapped by FarmCPU or BLINK model. The S6_3547612 was an unique QTL detected only by FarmCPU, S1_23581796 and S9_6774209 were two novel QTLs mapped by the BLINK model solely.

Seventeen unique QTLs were associated with PBN, 11, 1, 6, and 5 QTLs were identified by GLM, MLM, FarmCPU, BLINK methods (Supplementary Table 3 and Supplementary Fig. 6). S3_1337624 was a significant QTL detected by GLM, MLM, and BLINK with a *p*-value of 4.63E-12, 1.35E-08, and 5.08E-16. A previously reported gene *TAD1* located only 6.60 kb upstream of this locus. *TAD1* is an activator of the anaphase-promoting complex/cyclosome (APC/C) complex. The *tad1* mutant exhibited a reduced plant height and increased tiller number compared to the wild type [42]. The distance between S5_1405289 and S5_1408688 was merely 3.40 kb, which could be considered as a single locus. This locus was detected by GLM, FarmCPU, and BLINK. *OsSIZ1* was only 18.25 kb away from this QTL. Loss-of-function *ossiz1* mutation presented a significant decrease in root length, plant height, biomass, panicle length, number of primary rachis branches, and 1000-grain weight [43]. S9_19466707 is another QTL discovered by the GLM method with overlapped with previously reported genes *LGD1* [44]. *LGD1*-RNAi-4 and *LGD1*-RNAi-17 showed reduced tiller number, panicle length, the number of primary panicle branches, the number of grains per plant, and seed set percentages in comparison with wild type [44]. Through comparing the results with the Q-TARO database, the S4_31385963 was remapped by a previous study associated with the number of primary branches per panicle (Supplementary Table 3). Twelve novel QTLs were detected in the current study associated with PBN with four different methods.

Fifteen unique QTLs were significantly associated with PL in the current study. Six of them were identified by the GLM method and none of them was detected by MLM. In addition, 10 of them were detected by FarmCPU, and three were identified by the BLINK method (Supplementary Table 3 and Supplementary Fig. 7). Notably, S10_13359353 was discovered by GLM and FarmCPU simultaneously with the *p*-value of 3.80E-08 and 9.60E-09. This locus was nearby the reported gene *OsBRD2*, which is brassinosteroid (BR) biosynthesis. The *brd2* mutation showed a reduced plant height, shortened leaf sheaths, malformed panicles, reduced numbers of spikelets and rachis branches, and shorter first and second rachis branches compared to the wild type [45]. In addition, QTL S7_19447373 shared the region with a QTL *sp2(t)* for panicle length.

As for PN, a total of 17 QTLs were identified. Among them, 11, 1, 8, and 3 were detected by GLM, MLM, FarmCPU, and BLINK, respectively (Supplementary Table 3 and Supplementary Fig. 8). Three loci (S6_28002112, S7_25328075, and S9_16361023) were remapped to reported genes. The S6_28002112 was detected by GLM, FarmCPU, and BLINK with the *p*-value of 3.50E-11, 1.73E-08, and 4.58E-13, just 107.13 kb away from a previously reported gene *DEP3* [39]. The S7_25328075 and S9_16361023 were only detected by GLM with the *p*-value of 5.13E-10 and 6.13E-11, which are consistent with the reported gene *EP2/SRS1* and *DEP1*. The *EP2/SRS1* is predominantly expressed in vascular bundles, and the protein is localized to the endoplasmic reticulum [46]. The *srs1* mutants showed significantly reduced panicle number per plant compared to wild type and the loss-of-function also causes reduced grain length, width, thickness, as well as grain weight [47]. The *DEP1* encodes a phosphatidylethanolamine-binding protein (PEPB) like domain protein, which is highly similar to the N-terminus of *GS3*. NIL-*dep1* (gain-of-function mutation) represented a greater number of grains per panicle, shorter inflorescence internodes, and a greater number of both primary and secondary panicle branches compared to NIL-*DEP1* [48]*.* Besides, S2_24094693 shared the same region with *qPN2*, which was associated with panicle number [49]. Plus, six novel QTLs were identified by GLM, three of them were also detected by FarmCPU or BLINK method. Four and one unique new QTLs were discovered by FarmCPU and BLINK, separately.

To explore potential genes that may regulate TSNP, EGNP, GNP, PNB, PL, and PN in rice, we extracted genes in the local LD block. The detailed information of genes in the block was listed in Supplementary Table 4.

### Mining putative genes controlling the number of grain number per panicle

**Fig. 3:**
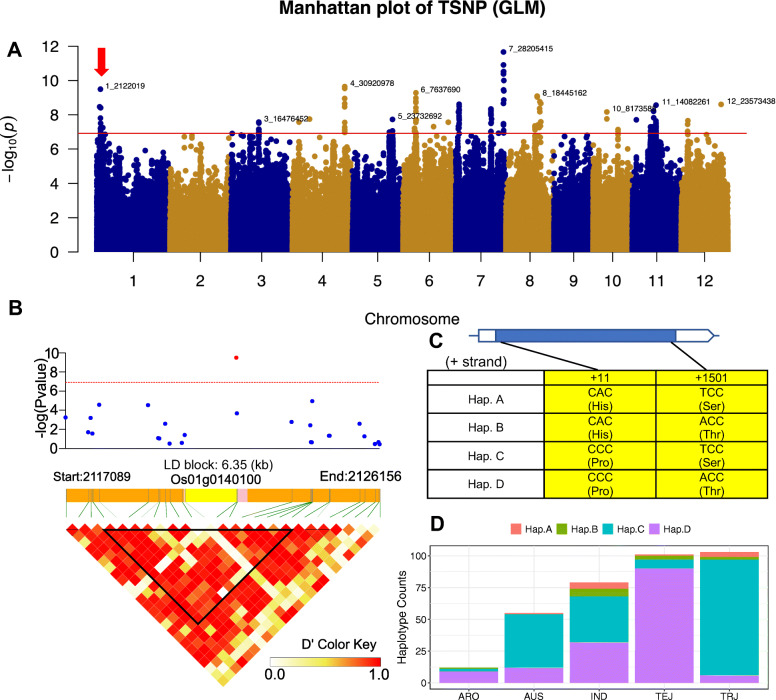
Identification of candidate gene associated with total spikelets number per panicle (TSNP). **a** Manhattan plot of TSNP with general linear model method. The red arrow indicates the interested QTL (S1_2122019) region. **b** Zoom in Manhattan plots of S1_2122019 and linkage disequilibrium (LD) heatmap (bottom). LD block was defined by the solid spine method. The gene structure is shown in the middle, the yellow, pink, and orange color indicate coding sequence (CDS), untranslated region (UTR), and intergenic regions, respectively. **c** Haplotype analysis of two non-synonymous SNPs in *Os01g0140100*. **d** Haplotype distribution in the different subgroups of the RDP1

A total of 65, 215, 91, 42, 11, and 25 genes are located in the LD block for TSNP, GNP, EGNP, PBN, PL, and PN traits, respectively, from GWAS analyses (Supplementary Table 3). Then, we compared these genes to the results detected by GPWAS. A total of 6 (TSNP), 4 (GNP), 35(EGNP), 4 (PBN), and 4 (PN) genes were overlapped from the two distinct studies (Supplementary Table 5). Strikingly, we found a gene (*Os01g0140100*) was detected by GWAS (*p*-value = 3.21E-10) and GPWAS (*p*-value = 9.01E-19) related to TSNP simultaneously. In the GWAS analysis, QTL (S1_2122019) was identified significantly associated with TSNP using the GLM method (Fig. 3a). Then a 6.35 LD block (Fig. 3b) was defined by the solid spine method. A unique gene (*Os01g0140100*) is located in this region. Besides, *Os01g0140100* was detected to be related to PN, TSNP, DHULGRLG, and EGNP traits by GPWAS (Supplementary Table 2). A total of three SNPs (1_2120529, 1_2122019, and 2,122,036) were detected in this gene, and two (1_2120529 and 1_2122019) of them could cause missense mutations identified by SnpEFF software (Supplementary Table 7). The first SNP locates on the eleventh coding sequence of *Os01g0140100*, changed nucleotide from C to A that resulted in the fourth amino acid altered from Proline (Pro) to Histidine (His). The second SNP posits at the 1501^st^ place of  the *Os01g0140100* coding sequence, the variation of SNP (A to T) led to the mutation of the 501^st^ amino acid from Threonine (Thr) to Serine (Ser). Besides, we searched the promoter of this gene on PlantPromoterDB website (http://ppdb.agr.gifu-u.ac.jp/ppdb/cgi-bin/index.cgi). The results showed that two Y-patch promoter elements located in the upstream 2000 bp (Supplementary Table 8), while no SNP was found in the two promoter elements. Therefore, we defined four types of haplotypes (Hap.A [His4Ser501], Hap. B [His4Thr501], Hap. C [Pro4Ser501], and Hap. D [Pro4Thr501]) based on the two non-synonymous SNPs (Fig. 3c), the heterozygous individuals were removed due to failed classification. Then, we compared the distribution of four haplotypes in ARO, AUS, IND, TEJ, and TRJ subpopulations (Fig. 3d). In general, Hap. C and Hap. D were predominant haplotypes in the RDP1 population, with haplotype frequency of 50.86% (178/350) and 42.57% (149/350), separately. In the ARO subpopulation, only three haplotypes (Hap.B, Hap. C, and Hap.D) were observed. The Hap. B didn't exist in the AUS subgroup. The percentages of Hap. C and Hap. D in IND, TEJ, and TRJ were significantly diversified. The ratio of Hap. C (45.57%) and Hap. D (40.51%) was similar in IND. Notably, the percentage of Hap. D in the TEJ subpopulation reached 89.11%, accounting for barely 5.83% in TRJ. In contrast, the ratio of Hap. C in the TRJ subgroup achieved 88.35%, while the value in TEJ was only 6.93% (Supplementary Table 6). Such significant differences in the two *Japonica* rice subpopulations (TEJ and TRJ) suggested the distinct evolutionary patterns, where intensive studies needed to address the mechanisms. Particularly, *OsMYB80* was reported to mediate anther development and pollen fertility by targeting multiple biological pathways [50], which could directly regulate *Os01g0140100* revealed by yeast one-hybrid assay. All the above hinted that *Os01g0140100* is a candidate gene regulating spikelets per panicle in rice.

**Fig. 4:**
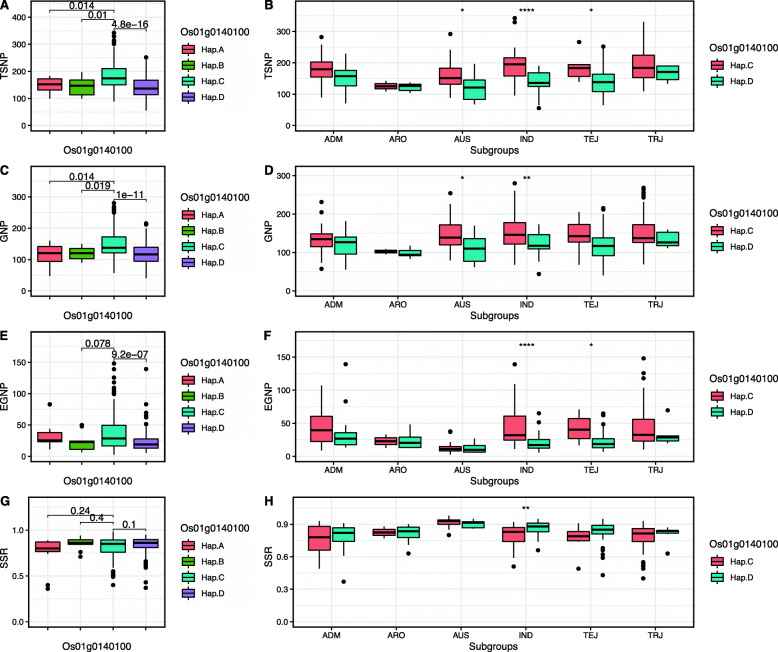
Boxplot of the 4 traits phenotypic variation in the whole population and subgroups. **a** Total spikelets number per panicle (TSNP) in the whole population. **b** TSNP in subgroups. **c** Grain number per panicle (GNP) in the whole population. **d** GNP in subgroups. **e** Empty grain number per panicle (EGNP) in the whole population. **f** EGNP in subgroups. **g** Seed setting rate (SSR) in the whole population. **h** SSR in subgroups. The bold lines in the middle represent the median values, the box edges represent the 0.25 and 0.75 quantiles, whiskers represent 1.5 times the interquartile range of the trait data, and points represent outliers. Differences between haplotypes were statistically analyzed using Wilcoxon test by R (*: *p* < 0.05; **: *p* < 0.01; ***: *p* < 0.001; ****: *p* < 0.0001). The numbers of plants carrying Hap. **a**, Hap. **b**, Hap. **c**, and Hap. **d** are shown in Supplementary Table 6

Accordingly, we compared the phenotypic differences of TSNP between the four haplotypes within the whole population. The findings revealed significant differences between Hap. A and Hap. C, Hap. B and Hap. C, Hap.D and Hap. C with the *p*-value of 0.014, 0.01, and 4.8E-16, respectively, as for TSNP values (Fig. 4a). The results indicated that rice plants with Hap. C haplotype of *Os01g0140100* highly enhanced the number of spikelets per panicle in contrast to other haplotypes. Considering the number of the haplotypes in each subpopulation, we next compared the differences between Hap. C and Hap. D of the total number of spikelets per panicle for different subgroups and observed significant differences from the AUS, IND, and TEJ subgroups (Fig. 4b). Moreover, we also performed comparisons in GNP (Fig. 4c and d) and EGNP (Fig. 4e and f), and similar results were obtained. Then, we calculated the seed setting rate (SSR) through dividing GNP by TSNP, and no distinct difference was found in the whole population (Fig. 4g). On the contrary, there was a significant difference in IND subgroups with a *p*-value at 0.01 level (Fig. 4h).

Furthermore, we analyzed the gene expressing pattern of *Os01g0140100* in 9 different tissues. We found *Os01g0140100* highly expresses in pre-emergence inflorescence, seed-5 DAP (seed 5 Days After Pollination), and pistil tissues. While in the anther, endosperm, and leaves, the expressing level is relatively lower than in the other tissues (Supplementary Fig. 9).

## Discussion

In this study, four different methods of GWAS were used to identify the QTLs associated with TSNP, GNP, EGNP, PBN, PN, and PL. The results showed that GLM identified more QTLs compared to MLM, FarmCPU, and BLINK. However, the GLM method employs the first three PCs as covariates, which may have a higher rate of false-positive. MLM uses both PCs and kinship matrix to reduce the false-positive rate, which sometimes causes overcorrection and could result in a reduced detecting power. Only a single QTL was discovered for TSNP, EGNP, PBN, and PN with MLM, and none QTL was identified for GNP and PL. FarmCPU and BLINK are two alternative methods to detect QTLs, which have higher statistical power and less false positives. *EUI1* and *OsCOL13* were simultaneously detected to be associate with TSNP by GLM and FarmCPU. *EUI1* and *OsPFPB* were identified related to GNP using GLM and FarmCPU. *OsBOR4* was discovered related to EGNP by GLM and FarmCPU. All of the above verified the power of GLM and FarmCPU. Meanwhile, *OsERF77*, *OsCOL13*, *OsOAT*, and *EP2* were recognized respectively related to TSNP, GNP, EGNP, and PN just by the GLM method. These three cases implied that although several alternative models were developed, the statistical power of GLM should not be ignored.

A total of 30 QTLs identified in the current study shared the same region as the previous study (Supplementary Table 3). In which, 20 genes and 12 QTLs were reported in GWAS or bi-parental populations. Through comparing the candidate genes mapped by GWAS and GPWAS, we identified 23 candidate genes related to rice panicle architectures, with 6, 4, 5, 4, and 4 associated with TSNP, GNP, EGNP, PBN, and PN, respectively. Among these candidate genes, all of them were identified by the GLM method except for the *Os07g0669700* gene. These results indicated that the GPWAS has more consistent results with the GLM method. Moreover, some of the homologous of the co-mapped genes have been reported related to grain number or yield in rice. On chromosome 7, a candidate gene (*Os07g0669700*) was identified related to TSNP by GWAS which was a potassium transporter 7 (*OsHAK7*). Besides, this gene was also associated with TSNP, PBN, DHULGRLG, FLFWD, DHULGRWD, AWNPLU using GPWAS. A homologous *Os04g0401700* (*OsHAK1*) has been reported regulating yield in rice. The loss-of-function mutant *oshak1* showed a significant decrease in K concentration, reduced length of root and shoot, grain yield, panicle length, and seed setting rate compared to wild type [51]. The *Os08g0159900* (*OsRH42*) gene is a homologous gene to *Os08g0416100* with an E-value of 2E-58. The *OsRH42* RNA*i* knockdown and overexpression lines were reported to display reduced panicle number, tiller number, panicle length, fewer seeds, and lighter seeds [52].

With the rapid development of automatic, multifunctional, and high-throughput phenotypic technologies, phenomics has become a new research area and provide a new scheme to develop new algorithms to study the relationship between markers and traits. Genome-Phenome Wide Association Study is recently designed to discover the relationship between genotype and multi-trait and has been employed to *Arabidopsis* and maize successfully [27]. This method focuses on the biological function to identify the potential multi-trait associated gene models. In addition to the overlapped gene *Os01g0140100* associated with TSNP identified by both GWAS and GPWAS*, OsROS1* and *OsCOM1* were another two genes discovered by GPWAS that related to EGNP, TSNP, GNP, and other traits, which were in line with the previous study [30, 31]. Besides, *Os01g0801700* (*OsGCD1*) was identified by GPWAS that related to HULGRWD, PTHT, DHULPROTCN, PL, FLFLG, HULGRVOL, DHULGRLG, TSNP, and LFLPUBES. This gene has been reported to play a vital role in rice fertility [53]. *Os02g0809800* (*OsPHO1;2*) is a phosphate transporter associated with HULGRWD, PTHT, FLFWD, DHULGRCL, PBN, PL, and TSNP according to GPWAS. The *ospho1;2* mutant showed significantly lower plant height, decreased panicle number, reduced grain number per panicle, declined 1000-grain weight, and yield per plant in rice [54]. Additionally, *WX1* (*Os06g0133000*) [55] is a well-studied gene controlling starch synthesis in rice. Using the GPWAS algorithm, this gene was found to be tightly (*p*-value = 4.14E-52) associated with AMYCN, which is consistent with previous studies. On the other hand, there are some limitations in GPWAS. Firstly, the GPWAS only considers the variant within genes, which ignores much potentially useful information on intergenic regions, such as the transposable elements (TE) and conserved non-coding sequence (CNS) regions. Secondly, the demand for SNP numbers is relatively higher than GWAS to cover more genes. In this study, we used a total of 411,066 SNPs which covered 62.40% (23,623/37,860) genes in rice. Taking *GS3* [16] and *GW5* [56] as an example, *GS3* is a well-studied gene controlling grain length and grain size in rice, which is also identified to be associated with DHULGRLG, CULMHAB, and ALKDIG with a *p*-value of 2.73E-39 (data was not shown). However, there was no SNP found in the *GW5* gene, resulting in a failed association of *GW5* using GPWAS. Deep resequencing data rather than SNP array data could be more suitable for the GPWAS approach to capture as much as gene regions.

## Conclusions

Our study provides new insights into TSNP, GNP, EGNP, PBN, PL, and PN regulation in rice using both GWAS and GPWAS approaches. A novel candidate gene (*Os01g0140100*) was mapped associated with TSNP. Through defining the haplotypes based on the two non-synonymous SNPs, we found significant differences between the Hap. C and Hap. D in the whole population and subgroups. This finding lays the foundation for maker-based improvement in rice. In-depth studies are needed to validate the function of this gene.

## Methods

### Plant materials

The rice diversity panel 1 (RDP1) consists of 421 purified homozygous varieties [28] including indica (IND), aus (AUS), tropical japonica (TRJ), temperate japonica (TEJ), and aromatic (ARO) subgroups. Among them, 406 individuals have been genotyped by High-Density Rice Array [57], which was used in this study. The High-Density Rice Array (HDRA, 700 k SNPs) dataset for RDP1 was obtained from the Rice Diversity website (http://www.ricediversity.org/data/). The detailed information of the varieties was listed in Supplementary Table 1.

### Traits related to panicle architecture

A total of six traits were analyzed in the current study, including TSNP (total spikelets number per panicle), GNP (grain number per panicle), EGNP (empty grain number per panicle), PBN (primary branch number), PL (panicle length), and PN (panicle number per plant). All the traits were obtained from the USDA website (https://www.ars.usda.gov/southeast-area/stuttgart-ar/dale-bumpers-national-rice-research-center/docs/rice-diversity-panel-1-rdp1/).

### SNP dataset and population structure

Through comparing the samples of genotype and phenotype data, 406 individuals were overlapped and remained for GWAS analysis in total. Firstly, the genotype file was filtered by PLINK software [58] (minor allele frequencies ≥0.05 and integrity ≥0.6), and 411,066 SNPs were passed for further study. Sequentially, genotype imputation was performed for the remaining 411,066 SNPs with Beagle 5.0 [59]. The PCA and kinship were used to evaluate the population structure with GAPIT software.

### Genome-Phenome Wide Association Study (GPWAS) analysis

For GPWAS analysis, multiple phenotypes are prerequisites. Therefore, we downloaded all the available phenotypic data for PDP1 from the USDA website (https://www.ars.usda.gov/southeast-area/stuttgart-ar/dale-bumpers-national-rice-research-center/docs/rice-diversity-panel-1-rdp1/). Fifteen individuals were filtered out due to the severe missing of phenotypic data. Overall, 391 individuals (Supplementary Table 1) with 27 traits (AMYCN [Amylose], ALKDIG [Alkali spreading value], DHULPROTCN [Protein content], DTHD [Days to heading], PTHT [Plant height], FLFLG [Flag leaf length], FLFWD [Flag leaf width], PN [Panicle number per plant], PL [Panicle length], PBN [Primary branch number], TSNP [Total spikelets number per panicle], EGNP [Empty grain number per panicle], GNP [Grain number per panicle], HULGRLG [Seed length], HULGRWD [Seed width], HULGRLGWDRO [Seed length/width ratio], HULGRVOL [Seed volume], HHULGRWT [100-Seed weight], DHULGRLG [Grain length], DHULGRWD [Grain width], DHULGRVOL [Grain volume], CULMHAB [Culm habit], SDSH [Seed shattering], AWNPLU [Awns], LFLPUBES [Leaf pubescence], HULCL [Hull color], and DHULGRCL [Bran color]) were collected and the missing values of phenotypes were further imputed with the median value of each traits. Based on the annotation file (37,860 genes, 2020-06-03 version) downloaded from The Rice Annotation Project Database (RAP-DB), the SNPs were assigned into individual genes while those posited intergenic regions were removed. The longest genes were selected for further study if overlapping was found among those genes. The genome-phenome wide association study was performed by GPWAS software [27]. The first three PCs were used as the covariates to correct the population structures as well as in GWAS analysis. The top 10% of genes were extracted from GPWAS results after sorting the genes’ *p*-value from low to high for the next step. In the current study, we focused on TSNP, GNP, EGNP, PBN, PL, and PN traits. Thus, candidate genes related to these six traits were kept for further analysis. Fifty times of permutations were performed to calculate the false discovery rate (FDR).

### Genome-Wide Association Study (GWAS) analyses

GWAS was implemented among the 406 rice varieties in RDP1 with the 411,066 high-quality SNPs. Univariate GWAS methods (GLM and MLM) and multivariate GWAS methods (FarmCPU and BLINK) were employed to evaluate the trait-SNP associations for the six target traits (TSNP, GNP, EGNP, PBN, PL, and PN) using the Genomic Association and Prediction Integrated Tool (GAPIT) [60]. The first three principal components (PCs) were used as covariates to correct population structure due to subpopulations in RDP1. The genome-wide significant thresholds of the GWAS (*p*-value = 1.22E-07) was determined by 0.05/n (n is the number of markers) [61]. The Manhattan and QQ plots for GWAS were visualized using the R package ‘qqman’ [62]. LD blocks were defined with the Solid Spine (SS) method and LD heatmap surrounding peaks in the GWAS was constructed using “LDBlockShow” in the R package [63].

### Mining putative genes and annotation of SNPs

The QTLs provide important information for understanding the genes regulating the grain number per panicle in rice. To explore candidate genes responsible for each QTL, we defined local LD with the Solid Spine (SS) method [64] and extracted all genes in the blocks. Additionally, we compared the genes resulted from GPWAS with the results from GWAS to narrow down the candidate genes. Finally, the SnpEff software [65] was specified to annotate the effect of the variant for the overlapped genes between GWAS and GPWAS (Supplementary Table 7).

### Expression profile of candidate genes

The expression patterns (normalized in fragments per kilobase of exon per million mapped reads [FPKM]) of a putative gene in eight tissues (anther, embryo, endosperm, leaves, pistil, inflorescence, and seed) were downloaded from the Rice Genome Annotation Project website (http://rice.plantbiology.msu.edu/index.shtml) and transformed with Log_2_ (FPKM+ 1).

## Supplementary Information

**Additional file 1: Figure S1.** The kinship plot of 406 accessions from RDP1. **Figure S2.** The distribution of SNP numbers in genes. **Figure S3.** Genome-wide association analysis for total spikelets number per panicle (TSNP) with general linear model (GLM), mixed linear models (MLM), Fixed and random model Circulating Probability Unification (FarmCPU), and Bayesian-information and Linkage-disequilibrium Iteratively Nested Keyway (BLINK) methods (left). Quantile-quantile plot of each model (right). Red texts indicate reported genes, blue texts indicate candidate genes. The horizontal solid red line indicates the Bonferroni-corrected significance threshold at -log_10_(P) = 6.91. **Figure S4.** Genome-wide association analysis for grain number per panicle (GNP) with general linear model (GLM), mixed linear models (MLM), Fixed and random model Circulating Probability Unification (FarmCPU), and Bayesian-information and Linkage-disequilibrium Iteratively Nested Keyway (BLINK) methods (left). Red texts indicate reported genes. The horizontal solid red line indicates the Bonferroni-corrected significance threshold and -log_10_(P) = 6.91. **Figure S5.** Genome-wide association analysis for EGNP with general linear model (GLM), mixed linear models (MLM), Fixed and random model Circulating Probability Unification (FarmCPU), and Bayesian-information and Linkage-disequilibrium Iteratively Nested Keyway (BLINK) methods (left). Quantile-quantile plot of each model (right). Red texts indicate reported genes. The horizontal solid red line indicates the Bonferroni-corrected significance threshold at -log_10_(P) = 6.91. **Figure S6.** Genome-wide association analysis for PBN with general linear model (GLM), mixed linear models (MLM), Fixed and random model Circulating Probability Unification (FarmCPU), and Bayesian-information and Linkage-disequilibrium Iteratively Nested Keyway (BLINK) methods (left). Quantile-quantile plot of each model (right). Red texts indicate reported genes. The horizontal solid red line indicates the Bonferroni-corrected significance threshold at -log_10_(P) = 6.91. **Figure S7.** Genome-wide association analysis for PL with general linear model (GLM), mixed linear models (MLM), Fixed and random model Circulating Probability Unification (FarmCPU), and Bayesian-information and Linkage-disequilibrium Iteratively Nested Keyway (BLINK) methods (left). Quantile-quantile plot of each model (right). Red texts indicate reported genes. The horizontal solid red line indicates the Bonferroni-corrected significance threshold at -log_10_(P) = 6.91. **Figure S8.** Genome-wide association analysis for PN with general linear model (GLM), mixed linear models (MLM), Fixed and random model Circulating Probability Unification (FarmCPU), and Bayesian-information and Linkage-disequilibrium Iteratively Nested Keyway (BLINK) methods (left). Quantile-quantile plot of each model (right). Red texts indicate reported genes. The horizontal solid red line indicates the Bonferroni-corrected significance threshold at -log_10_(P) = 6.91. **Figure S9.** Expression pattern of *Os01g0140100* in different tissues. Anther: Anther; Embryo-25 DAP: Embryo 25 Days After Pollination; Endosperm-25 DAP: Endosperm 25 Days After Pollination; Leaves-20 days: 20 Day Leaves; Pistil: Pistil; Pre-emergence inflorescence: Early Inflorescence; Post-emergence inflorescence: Emerging Inflorescence; Seed-5 DAP: Seed 5 Days After Pollination; Seed-10 DAP: Seed 10 Days After Pollination.

**Additional file 2: Table S1.** The sample list and structure information of 406 accessions. **Table S2.** Significant genes detected using GPWAS and the phenotypes selected for each gene model. **Table S3.** The QTLs associated with TSNP, GNP, EGNP, PBN, PL, and PN with four different methods. **Table S4.** List of genes located in the LD block for TSNP, GNP, EGNP, PBN, PL, and PN. **Table S5.** Overlapped genes related to TSNP, GNP, EGNP, PBN, PN, and PL using GPWAS and GWAS methods. **Table S6.** Distribution of four haplotypes in subgroups. **Table S7.** Annotation of all SNPs located in the overlapped genes using SnpEff software. **Table S8.** Promoter elements anslysis of gene *Os01g0140100.*

## Data Availability

The genotype datasets analyzed during the current study are available in the GEO database (accession ID: GSE71553), the phenotype traits analyzed are available in website https://www.ars.usda.gov/southeast-area/stuttgart-ar/dale-bumpers-national-rice-research-center/docs/rice-diversity-panel-1-rdp1/.

## Abbreviations

RDP1: Rice Diversity Panel 1
SNP: Single-nucleotide polymorphism
GWAS: Genome-wide association study
TSNP: Total spikelets number per panicle
GNP: Grain number per panicle
EGNP: Empty grain number per panicle
PBN: Primary branch number
PL: Panicle length
PN: Panicle number per plant
GLM: General linear model
MLM: Mixed linear models
FarmCPU: Fixed and random model Circulating Probability Unification
BLINK: Bayesian-information and Linkage-disequilibrium Iteratively Nested Keyway
GPWAS: Genome-phenome wide association study
IND: Indica
AUS: Aus
TRJ: Tropical japonica
TEJ: Temperate japonica
ARO: Aromatic
HDRA: High-Density Rice Array
GAPIT: Genomic Association and Prediction Integrated Tool
SS: Solid Spine
AMYCN: Amylose
ALKDIG: Alkali spreading value
DHULPROTCN: Protein content
DTHD: Days to heading
PTHT: Plant height
FLFLG: Flag leaf length
FLFWD: Flag leaf width
HULGRLG: Seed length
HULGRWD: Seed width
HULGRLGWDRO: Seed length/width ratio
HULGRVOL: Seed volume
HHULGRWT: 100-Seed weight
DHULGRLG: Grain length
DHULGRWD: Grain width
DHULGRVOL: Grain volume
CULMHAB: Culm habit
SDSH: Seed shattering
AWNPLU: Awns
LFLPUBES: Leaf pubescence
HULCL: Hull color
DHULGRCL: Bran color
RAP-DB: Rice Annotation Project Database
FDR: False discovery rate
FPKM: Fragments per kilobase of exon per million mapped reads
PCA: Principal component analysis
PCs: Principal components
LD: Linkage disequilibrium
BIC: Bayesian Information Content
SSR: Seed setting rate
