# Correction to: uncovering the genetic mechanisms regulating panicle architecture in rice with GPWAS and GWAS

**DOI:** 10.1186/s12864-021-07530-4

**Published:** 2021-03-23

**Authors:** Hua Zhong, Shuai Liu, Xiaoxi Meng, Tong Sun, Yujuan Deng, Weilong Kong, Zhaohua Peng, Yangsheng Li

**Affiliations:** 1 State Key Laboratory of Hybrid Rice, Key Laboratory for Research and Utilization of Heterosis in Indica Rice, Ministry of Agriculture, College of Life Sciences, Wuhan University, Wuhan, China; 2 Department of Biochemistry, Molecular Biology, Entomology and Plant Pathology, Mississippi State University, Starkville, MS 39762 USA; 3 Department of Computer Science and Engineering, Experimental Teaching Center, Shijiazhuang University, Shijiazhuang, Hebei China

**Correction to: BMC Genomics 22, 86 (2021).**

**https://doi.org/10.1186/s12864-021-07391-x**

Following publication of the original article [1], the authors reported an error in the order of the grants in the Funding declaration. The correct Funding declaration is given below, and the original article has been updated.

**Funding.**

The study was supported by the National Key Research and Development Program of China (2016YFD0100400) and the National Special Key Project for Transgenic Breeding (Grant No. 2016ZX08001001). The funding agencies played no role in the design of the study, data collection, analysis, and interpretation or writing the manuscript.
