# Supplementary material for: Uncovering the genetic mechanisms regulating panicle architecture in rice with GPWAS and GWAS
Source: BMC Genomics. 2021 Jan 28;22:86. doi: 10.1186/s12864-021-07391-x (PMC7842007; doi:10.1186/s12864-021-07391-x)
Supplement: Supplementary file 1 — Additional file 1: Figure S1. The kinship plot of 406 accessions from RDP1. Figure S2. The distribution of SNP numbers in genes. Figure S3. Genome-wide association analysis for total spikelets number per panicle (TSNP) with general linear model (GLM), mixed linear models (MLM), Fixed and random model Circulating Probability Unification (FarmCPU), and Bayesian-information and Linkage-disequilibrium Iteratively Nested Keyway (BLINK) methods (left). Quantile-quantile plot of each model (right). Red texts indicate reported genes, blue texts indicate candidate genes. The horizontal solid red line indicates the Bonferroni-corrected significance threshold at -log10(P) = 6.91. Figure S4. Genome-wide association analysis for grain number per panicle (GNP) with general linear model (GLM), mixed linear models (MLM), Fixed and random model Circulating Probability Unification (FarmCPU), and Bayesian-information and Linkage-disequilibrium Iteratively Nested Keyway (BLINK) methods (left). Red texts indicate reported genes. The horizontal solid red line indicates the Bonferroni-corrected significance threshold and -log10(P) = 6.91. Figure S5. Genome-wide association analysis for EGNP with general linear model (GLM), mixed linear models (MLM), Fixed and random model Circulating Probability Unification (FarmCPU), and Bayesian-information and Linkage-disequilibrium Iteratively Nested Keyway (BLINK) methods (left). Quantile-quantile plot of each model (right). Red texts indicate reported genes. The horizontal solid red line indicates the Bonferroni-corrected significance threshold at -log10(P) = 6.91. Figure S6. Genome-wide association analysis for PBN with general linear model (GLM), mixed linear models (MLM), Fixed and random model Circulating Probability Unification (FarmCPU), and Bayesian-information and Linkage-disequilibrium Iteratively Nested Keyway (BLINK) methods (left). Quantile-quantile plot of each model (right). Red texts indicate reported genes. The horizontal solid red l [file 12864_2021_7391_MOESM1_ESM.pdf]

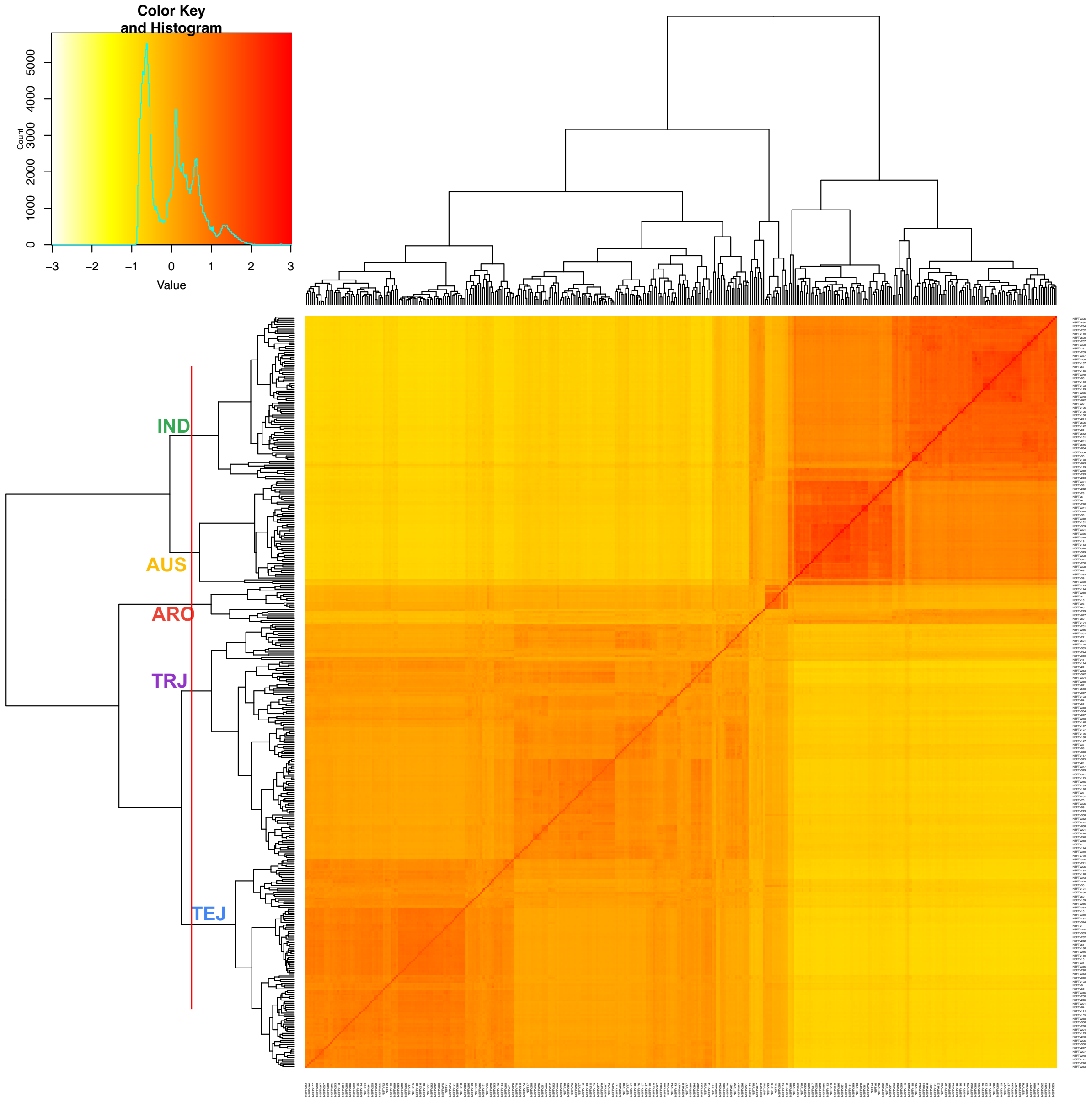

Supplementary Figure 1. The kinship plot of 406 accessions from RDP1.

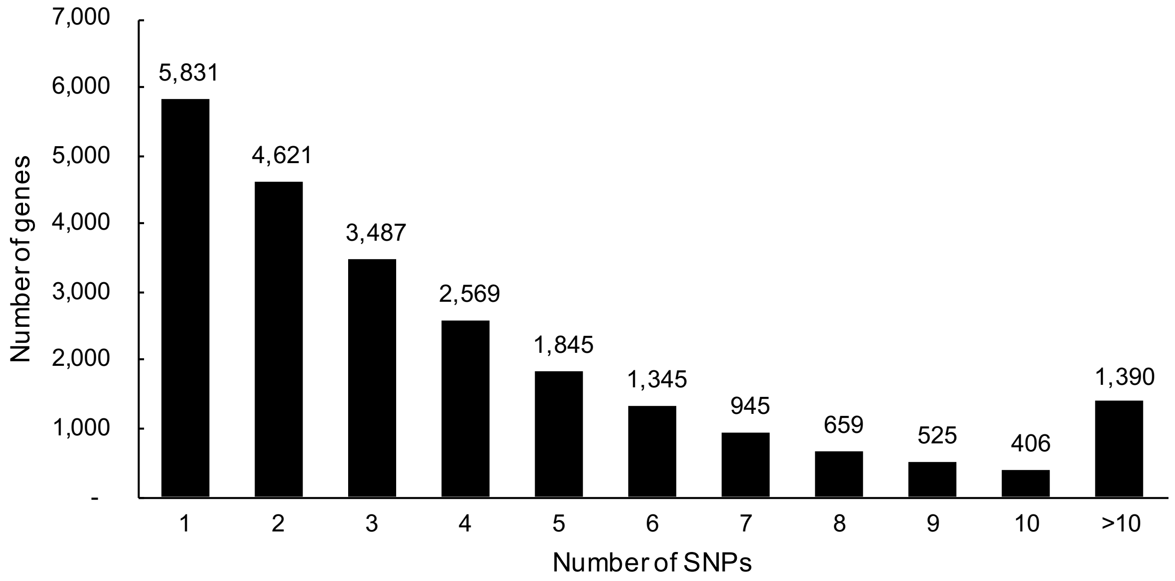

Supplementary Figure 2. The distribution of SNP number in genes.

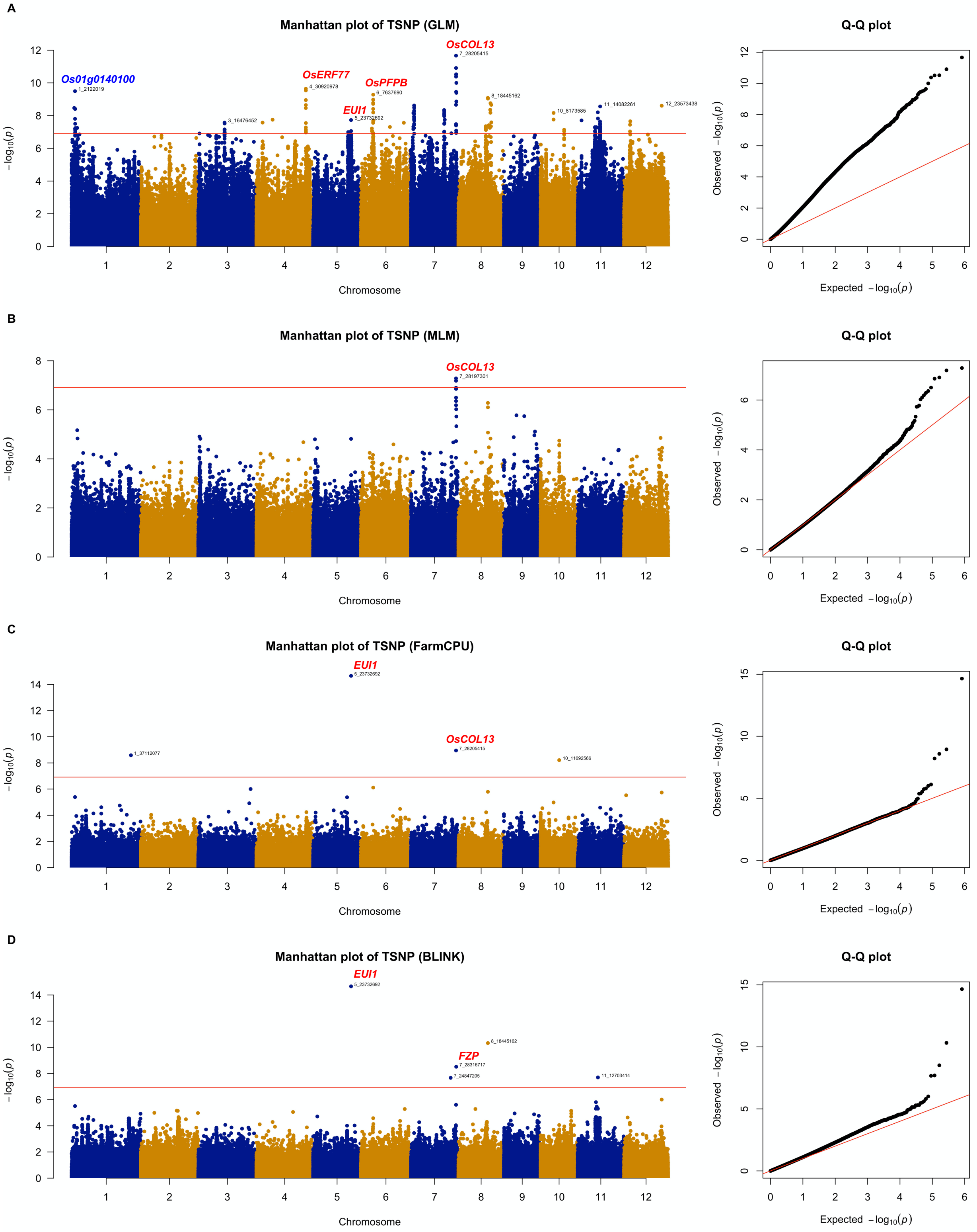

Supplementary Figure 3. Genome-wide association analysis for total spikelets number per panicle (TSNP) with general linear model (GLM), mixed linear models (MLM), Fixed and random model Circulating Probability Unification (FarmCPU), and Bayesian-information and Linkage-disequilibrium Iteratively Nested Keyway (BLINK) methods (left). Quantile-quantile plot of each model (right). Red texts indicate reported genes, blue texts indicate candidate genes. The horizontal solid red line indicates the Bonferroni-corrected significance threshold $-\log_{10}(P) = 6.91$.

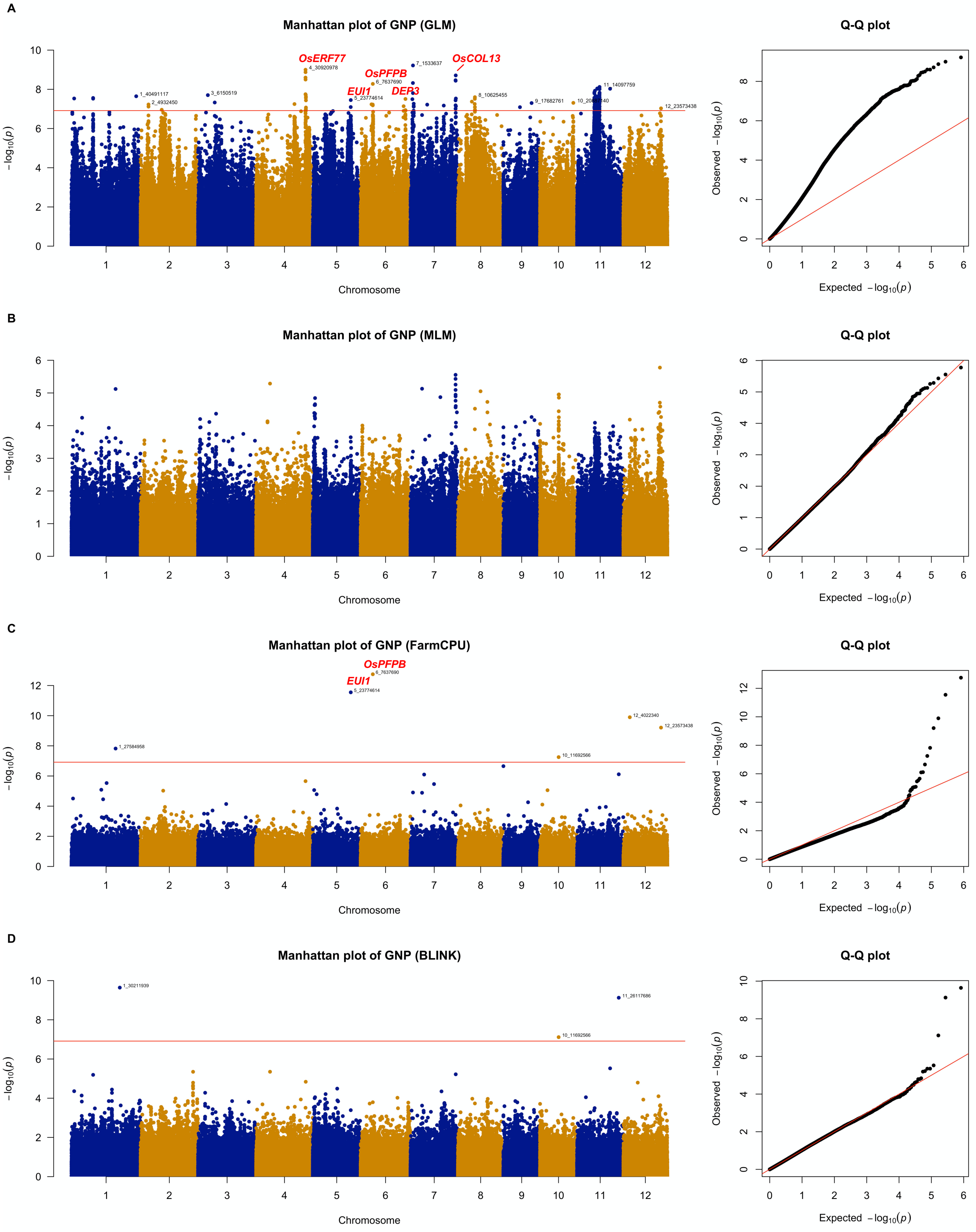

Supplementary Figure 4. Genome-wide association analysis for grain number per panicle (GNP) with general linear model (GLM), mixed linear models (MLM), Fixed and random model Circulating Probability Unification (FarmCPU), and Bayesian-information and Linkage-disequilibrium Iteratively Nested Keyway (BLINK) methods (left). Red texts indicate reported genes. The horizontal solid red line indicates the Bonferroni-corrected significance threshold -$\log_{10}$(P) = 6.91.

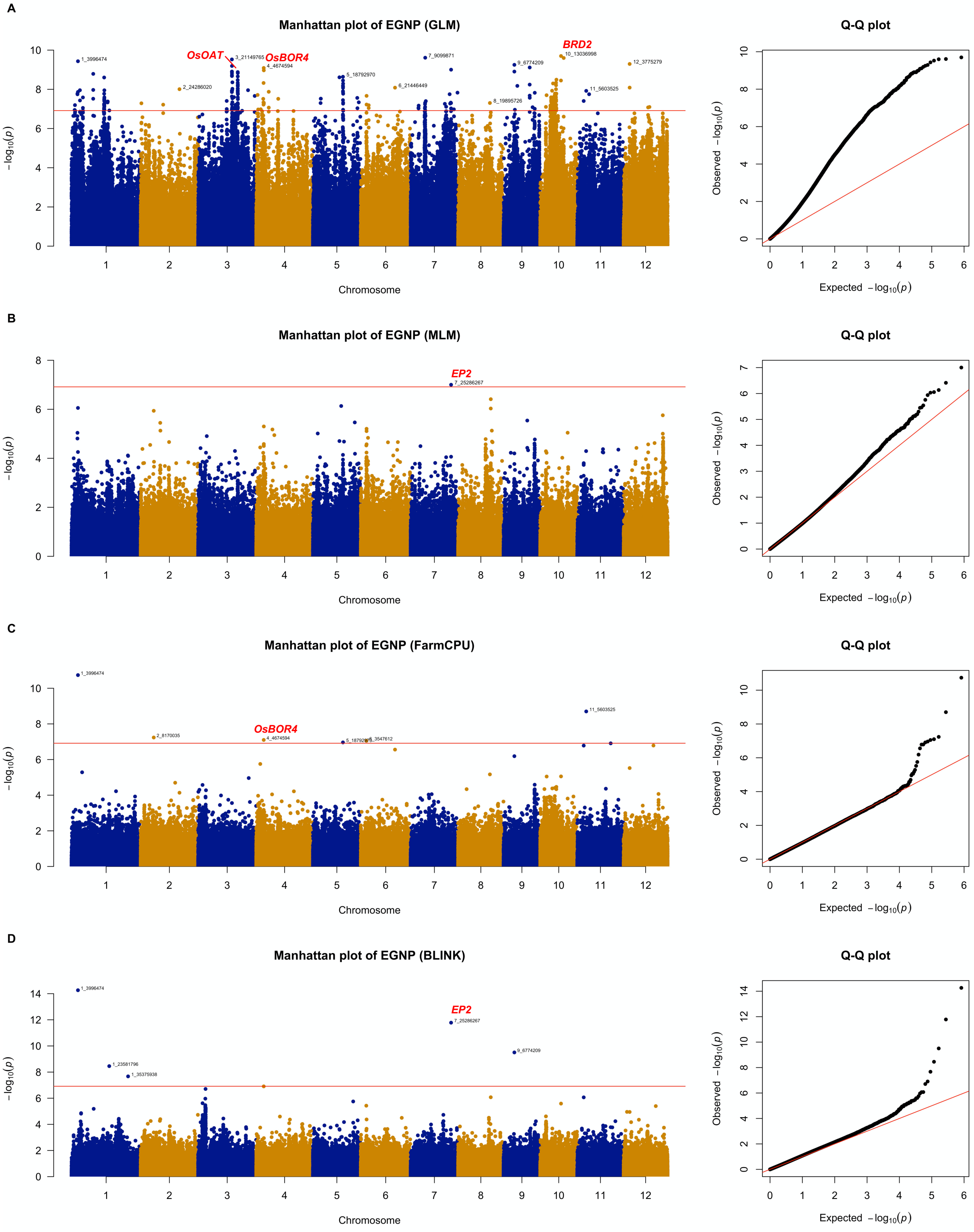

Supplementary Figure 5. Genome-wide association analysis for EGNP with general linear model (GLM), mixed linear models (MLM), Fixed and random model Circulating Probability Unification (FarmCPU), and Bayesian-information and Linkage-disequilibrium Iteratively Nested Keyway (BLINK) methods (left). Quantile-quantile plot of each model (right). Red texts indicate reported genes. The horizontal solid red line indicates the Bonferroni-corrected significance threshold $-\log_{10}(P) = 6.91$.

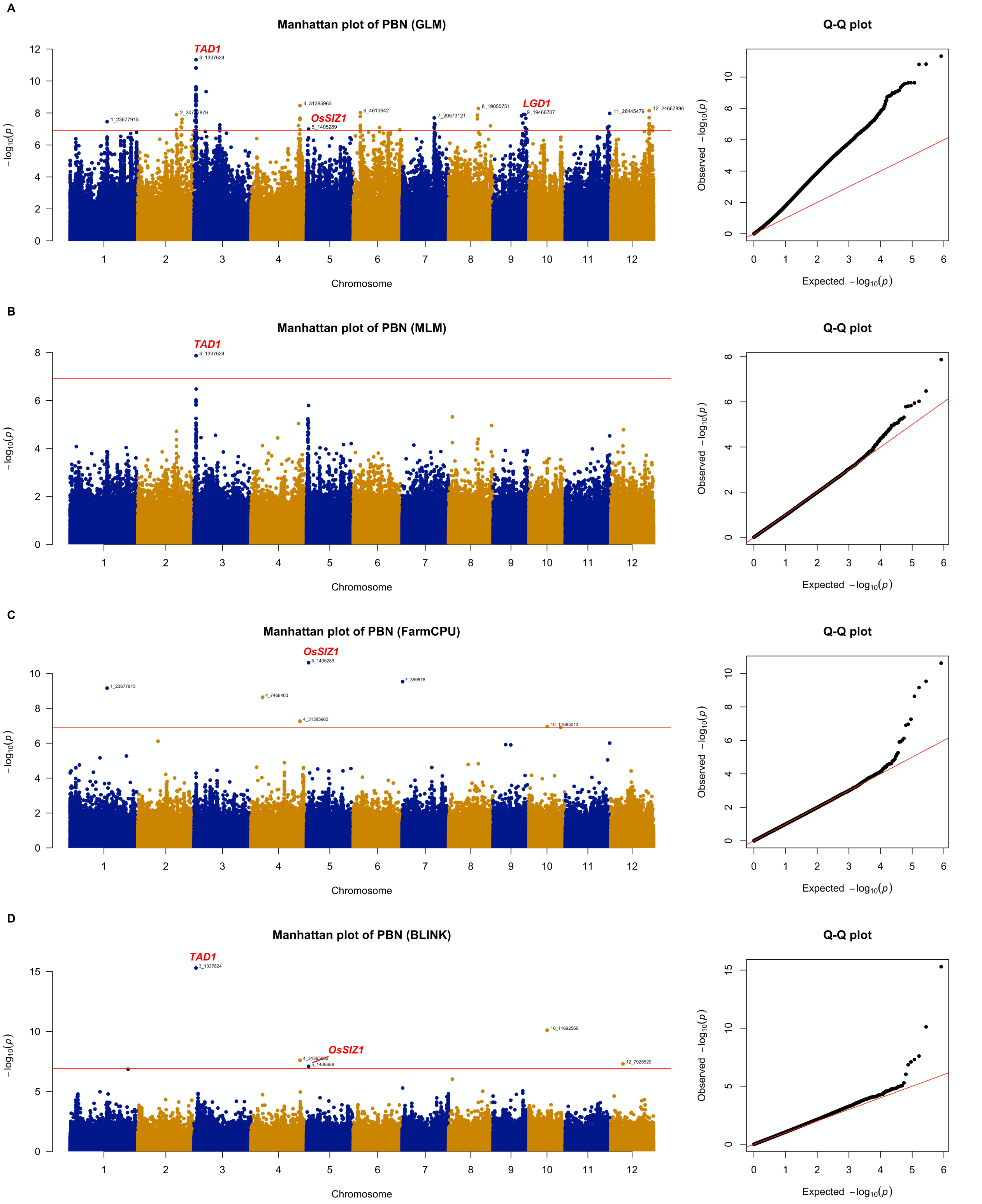

Supplementary Figure 6. Genome-wide association analysis for PBN with general linear model (GLM), mixed linear models (MLM), Fixed and random model Circulating Probability Unification (FarmCPU), and Bayesian-information and Linkage-disequilibrium Iteratively Nested Keyway (BLINK) methods (left). Quantile-quantile plot of each model (right). Red texts indicate reported genes. The horizontal solid red line indicates the Bonferroni-corrected significance threshold -$\log_{10}$(P) = 6.91.

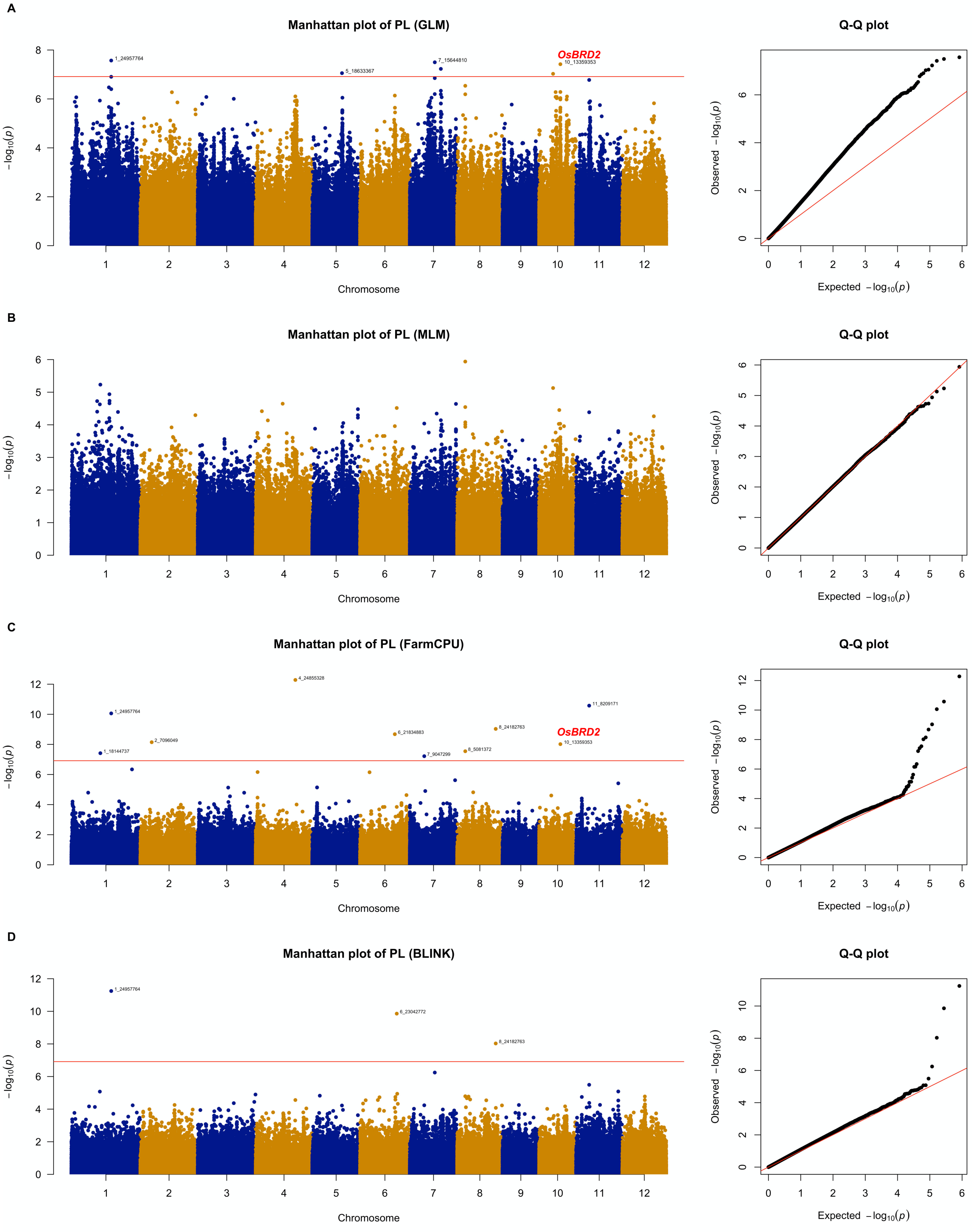

Supplementary Figure 7. Genome-wide association analysis for PL with general linear model (GLM), mixed linear models (MLM), Fixed and random model Circulating Probability Unification (FarmCPU), and Bayesian-information and Linkage-disequilibrium Iteratively Nested Keyway (BLINK) methods (left). Quantile-quantile plot of each model (right). Red texts indicate reported genes. The horizontal solid red line indicates the Bonferroni-corrected significance threshold $-\log_{10}(P) = 6.91$.

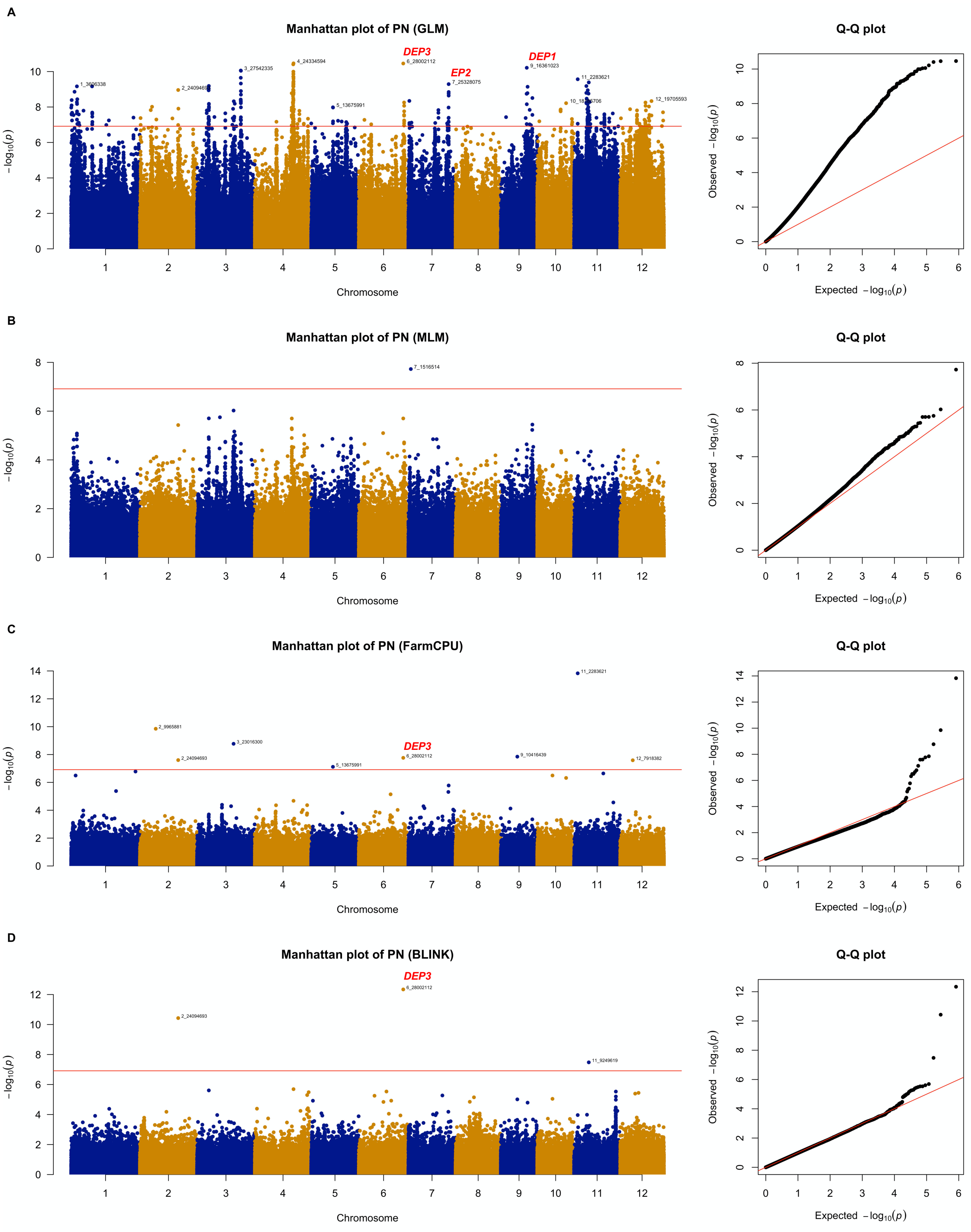

Supplementary Figure 8. Genome-wide association analysis for PN with general linear model (GLM), mixed linear models (MLM), Fixed and random model Circulating Probability Unification (FarmCPU), and Bayesian-information and Linkage-disequilibrium Iteratively Nested Keyway (BLINK) methods (left). Quantile-quantile plot of each model (right). Red texts indicate reported genes. The horizontal solid red line indicates the Bonferroni-corrected significance threshold -$\log_{10}$(P) = 6.91.

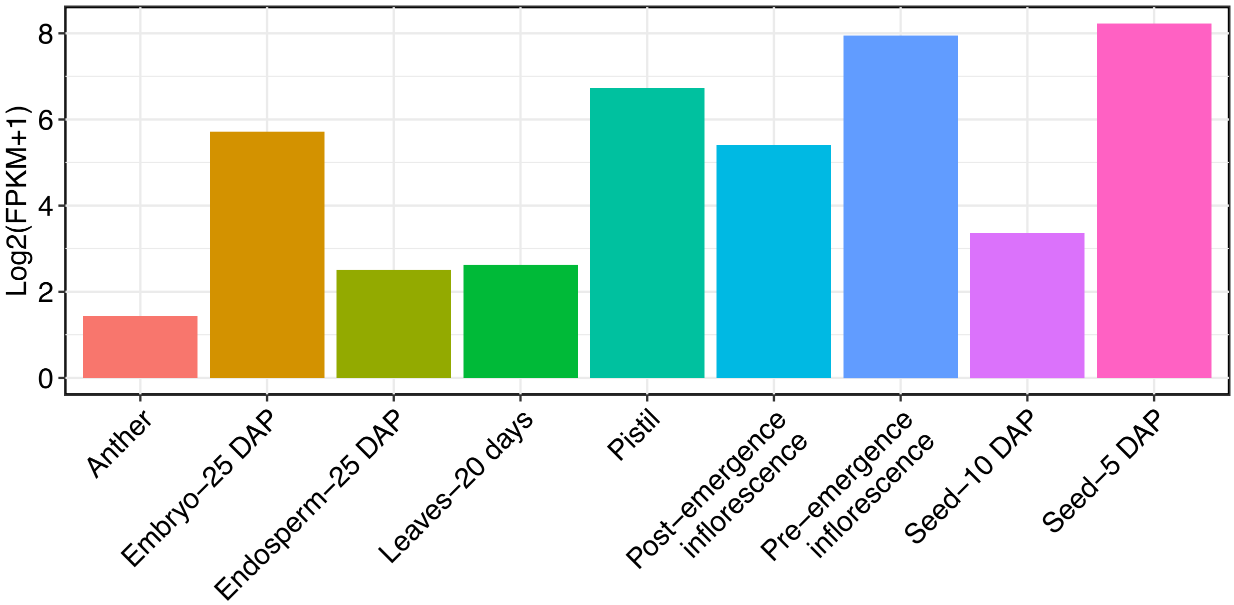

Supplementary Figure 9. Expression pattern of *Os01g0140100* in different tissues. Anther: Anther; Embryo-25 DAP: Embryo 25 Days After Pollination; Endosperm-25 DAP: Endosperm 25 Days After Pollination; Leaves-20 days: 20 Day Leaves; Pistil: Pistil; Pre-emergence inflorescence: Early Inflorescence; Post-emergence inflorescence: Emerging Inflorescence; Seed-5 DAP: Seed 5 Days After Pollination; Seed-10 DAP: Seed 10 Days After Pollination.
